# Real‐time *in vivo* dosimetry for SBRT prostate treatment using plastic scintillating dosimetry embedded in a rectal balloon: a case study

**DOI:** 10.1120/jacmp.v17i6.6508

**Published:** 2016-11-08

**Authors:** Justin L. Cantley, Chee‐Wai Cheng, Fredrick B. Jesseph, Tarun K. Podder, Valdir C. Colussi, Bryan J. Traughber, Lee E. Ponsky, Rodney J. Ellis

**Affiliations:** ^1^ Department of Radiation Oncology University Hospitals Cleveland OH USA; ^2^ Department of Urology University Hospitals Cleveland OH USA

**Keywords:** SBRT, *in vivo* dosimetry, plastic scintillator, real‐time dosimetry

## Abstract

A novel FDA approved *in vivo* dosimetry device system using plastic scintillating detectors placed in an endorectal balloon to provide real‐time *in vivo* dosimetry for prostatic rectal interface was tested for use with stereotactic body radiotherapy (SBRT). The system was used for the first time ever to measure dose during linear accelerator based SBRT. A single patient was treated with a total dose of 36.25 Gy given in 5 fractions. Delivered dose was measured for each treatment with the detectors placed against the anterior rectal wall near the prostate rectal interface. Measured doses showed varying degrees of agreement with computed/ planned doses, with average combined dose found to be within 6% of the expected dose. The variance between measurements is most likely due to uncertainty of the detector location, as well as variation in the placement of a new balloon prior to each fraction. Distance to agreement for the detectors was generally found to be within a few millimeters, which also suggested that the differences in measured and calculated doses were due to positional uncertainty of the detectors during the SBRT, which had sharp dose falloff near the penumbra along the rectal wall. Overall, the use of a real time *in vivo* dosimeter provided a level of safety and improved confidence in treatment delivery. We are evaluating the device further in an IRB‐approved prospective partial prostate SBRT trial, and believe further clinical investigations are warranted.

PACS number(s): 87.53.Bn, 87.53.Ly, 87.55.km

## I. INTRODUCTION

The patient was a 55‐year‐old male with localized prostate cancer treated with linear‐accelerator‐based stereotactic body radiation therapy (SBRT) to deliver 36.25 Gy in 5 fractions of 7.25 Gy per fraction. Due to the high‐stakes nature of SBRT, it is prudent to employ some type of treatment delivery verification. *In vivo* dosimetry is commonly used in external beam radiation therapy in order to detect major errors in treatment delivery, to assess how well the delivered dose matches the planned dose, to record the actual dose received, and to fulfill legal requirements.^1^, ^2^ The dose to the rectal wall is of interest as the institution has plans to move forward with a new clinical trial that will treat partial prostate with linear‐accelerator‐based SBRT and dose to the rectal wall is a particular concern of the trial. As such, *in vivo* dosimetry measurements were made of the anterior rectal wall using plastic scintillators. Plastic scintillators are well suited for *in vivo* dosimetry measurements as they are water‐equivalent; independent of angular incidence, dose rate, and energy; and have a linear relationship between dose deposited and light emitted^3^, ^4^ However, newer generations of plastic scintillating materials exhibit a temperature dependence not found in previously used materials.^5^, ^6^

## II. MATERIALS AND METHODS

The patient was a 55‐year‐old male with localized prostate cancer clinical stage T1cN0M0, Gleason 7(3+4) initial PSA 6.6 ng/mL Stage IIa with ECOG performance status of 0. He presented for consultation inquiring about advanced radiotherapy techniques including proton therapy and stereotactic radiosurgery. His main concerns were late effects of the radiotherapy for quality of life. After consultation with an urologist and radiation oncologist to discuss management, he elected linear‐accelerator‐based stereotactic body radiation therapy (SBRT) to deliver 36.25 Gy in 5 fractions of 7.25 Gy per fraction. Fiducial markers were placed in the operating room transperineally by his urologist, and at the time of fiducial marker placement a hydrogel device (SpaceOAR, Augmenix Inc. Waltham, ME) was placed to separate the prostate and the rectal wall to reduce the risk for rectal toxicity related to the radiation exposure.^(7)^

**Figure 1:**
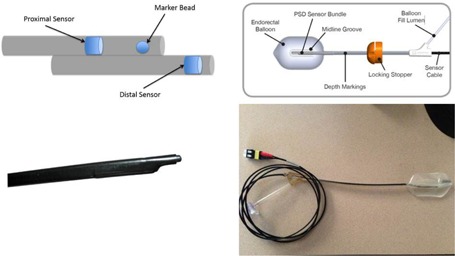
OARtrac system: (top left) schematic of the PSD sensors and the fiducial (marker bead); (top right) overhead schematic of the endorectal balloon with the PSD sensor connected. Note that the sensor is attached to the top side of the balloon. Bottom left: PSD sensor outside of the endorectal balloon. Bottom right: Overhead view of the PSD sensor inside of the endorectal balloon and the fiber optic cables that will connect to the CCD camera system. The sensor is attached to the top side of the balloon, which would be placed against the anterior rectal wall.

The OARtrac system (RadiaDyne, Houston, TX) is a new *in vivo* scintillation dosimetry system designed to measure rectal wall dose during prostate radiotherapy procedures. The OARtrac system uses a single‐use prostate immobilization endorectal balloon (ERB) embedded with two independent plastic scintillation radiation detectors that provide near real‐time dose verification for external beam irradiation of prostatic cancer.^(7)^ Two plastic scintillating detectors (PSDs) are installed on the anterior surface and along the length of an endorectal balloon (labeled as proximal and distal, respectively).^8^, ^9^, ^10^ These PSDs measure the dose at the prostatic rectal interface where the dose gradient is steep as the patient is being irradiated with megavoltage X‐rays. The rectal balloon reduces the motion of the prostate gland to a minimum while at the same time maintains a constant shape of the rectum; see Fig. 1 for more details about the system. Use of the system is simplified by system‐specific software. The system simply needs a few minutes to warm up, input the sensor used, and take a background measurement before each measurement. After each measurement, the user has the option to create a PDF report, and the measurement is saved in the system automatically.

The sensors were precalibrated at the University of Texas MD Anderson Cancer Center Dosimetry Laboratory, but did require an on‐site correction for SBRT treatments. During installation of the system, a dose verification test was performed to assess the accuracy of the pair of PSD detectors using a solid‐water phantom and the patient‐specific plan. These measurements were compared against the expected machine output under the same irradiation condition. To reduce the variability of detector response to radiation during the SBRT treatment, a total of five different PSDs pairs were placed sequentially in the solid‐water phantom and measured under the same conditions. Using the measured values, a single system adjustment was made to ensure the dose measurement accuracy. The PSD sensors used during patient treatment were then tested using the solid‐water phantom and the patient‐specific plan, which allowed for a controlled test with minimal positional uncertainty. Differences between the measured dose and the planned dose were found to be within 2% and 1%, on average, for the proximal and distal sensors respectively when using the solid‐water phantom. This was done to test the accuracy of the various sensors used for dose measurements during treatment.

For patient treatments, the system was used to measure real‐time dose delivered to the patient prostatic rectal interface for each fraction. The measured dose was compared to the computed dose to the rectal wall for SBRT from the treatment planning system (Pinnacle, Philips Healthcare, Madison, WI). The computed dose was found by creating a region of interest (ROI) and determining the mean dose to the ROI. PSD location was determined using the location of the fiducial in the sensor and the known distances between the fiducial and the sensors. In addition, fraction‐specific computed dose was found to compare with the measured dose. This was done using the cone‐beam CT (CBCT) taken between the two treatment arcs. This CBCT was chosen because it was thought to be the most representative of the total treatment fraction. The CBCT was exported to MIM (MIM Software, Cleveland, OH), a software that allowed the CBCT to be fused to the original treatment planning CT and the treatment planning dose transferred to the CBCT. Rigid registration was performed based upon fiducials in the prostate, as this was the method used during patient treatment since the distance between the anterior rectal wall and the prostate was minimal. Each of the system's ERB sensors included a fiducial between the two PSDs which was visible in each CBCT, and allowed a better approximation of the placement of the PSD sensors, which might suffer from interfractional positional variation. This information was used to determine a more appropriate predicted dose for each individual fraction.

For each treatment fraction, a new endorectal balloon (ERB) and sensor was used. Residual air was removed from the balloon to prevent gas pockets and it was then filled with water before insertion into the rectal cavity. The ERB was placed with lubricating gel with the PSD devices positioned to press on the anterior rectal wall to improve heterogeneity. The balloon was filled to a total of 40 cc of water to help immobilize the gland without exerting excess pressure against the prostate since the excess pressure could move the rectal wall closer to the PTV. Once the balloon was inserted and filled, it was retracted to hold against the anal sphincter. The external rectal stopper was locked onto the shaft of the balloon at the same distance from the tip each day for reproducibility to match the daily treatment distance from the anal verge. Radiation dose measured for each treatment fraction consisted of two treatment arcs and a CBCT taken between the two arcs. Any dose measured from the CBCT was subtracted from the final reading so that only the treatment dose was considered. The only change to the workflow of a typical prostate treatment using a rectal balloon was the presence of a physicist trained to use the OARtrac system. Setup of the system (machine warm‐up, connection of sensors, and background measurements) was able to be performed during patient positioning so that treatment was not prolonged or delayed.

At the time of the final consultation, informed consent was obtained to proceed with SBRT for the prostate gland using fiducial markers and hydrogel placement prior to treatment planning, as well as for daily use of the PSD and ERB device during treatment to record the *in vivo* rectal dose. While the hydrogel was placed to provide distance between the rectal wall and the prostate, the use of the PSD and ERB system was not only to measure rectal wall dose *in vivo*, but also to prevent prostate motion during the SBRT delivery to maximize the benefit from image guidance during the procedure in a complimentary fashion for each device to improve patient safety and treatment delivery. Utilizing both technologies allowed us to limit average rectal wall dose to 36.9% of prescribed daily dose to the prostate gland and verify this *in vivo*. All procedures were performed in accordance with the ethical standards set forth by the IRB committee and with the Helsinki Declaration of 1975, as revised in 2000.

## III. RESULTS

**Figure 2:**
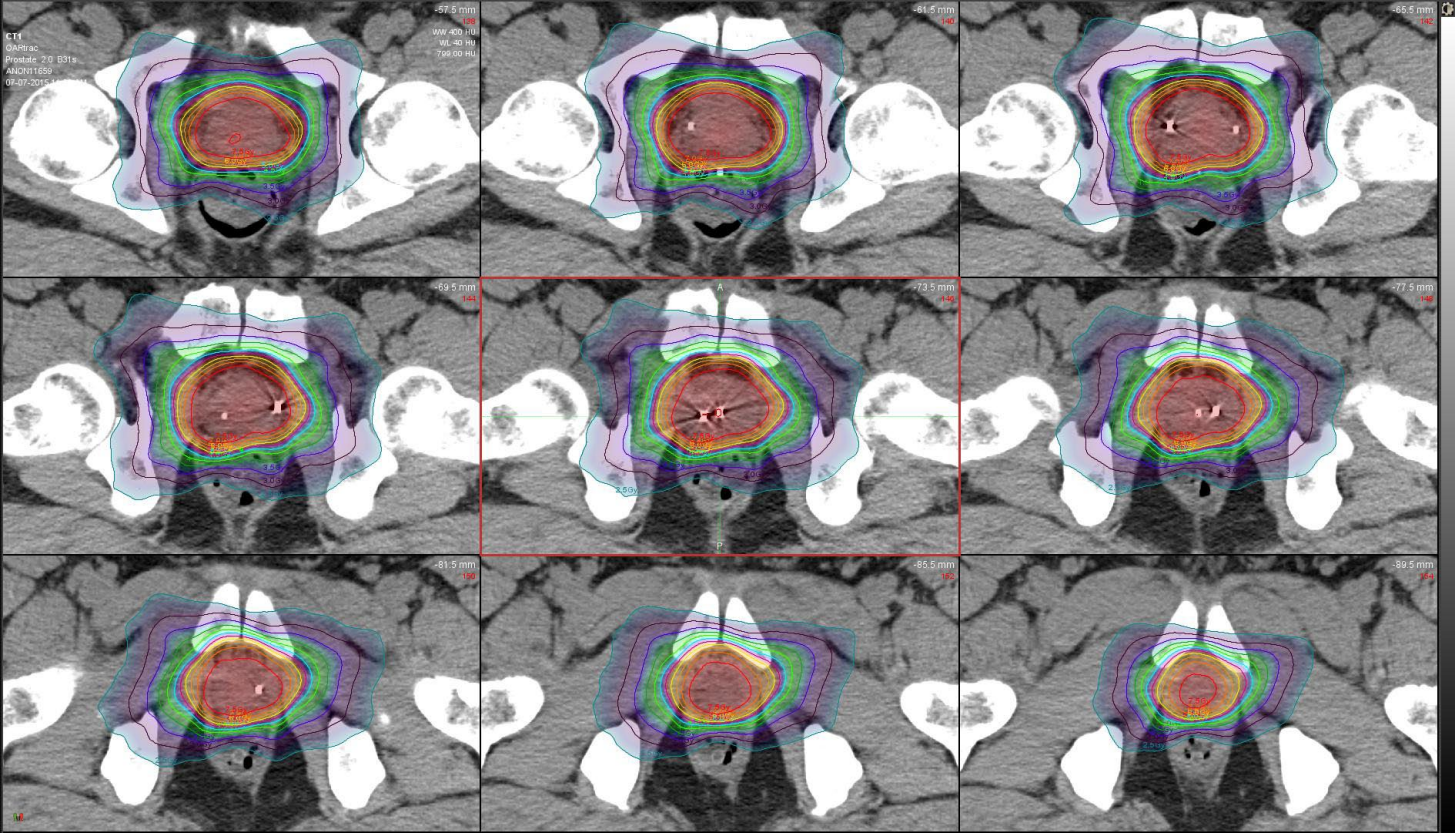
Axial view of the prostate with isodose lines for one fraction of the SBRT treatment. Each isodose line represents a change of 50 cGy. Slices moving superior to inferior from top left to bottom right.

**Figure 3:**
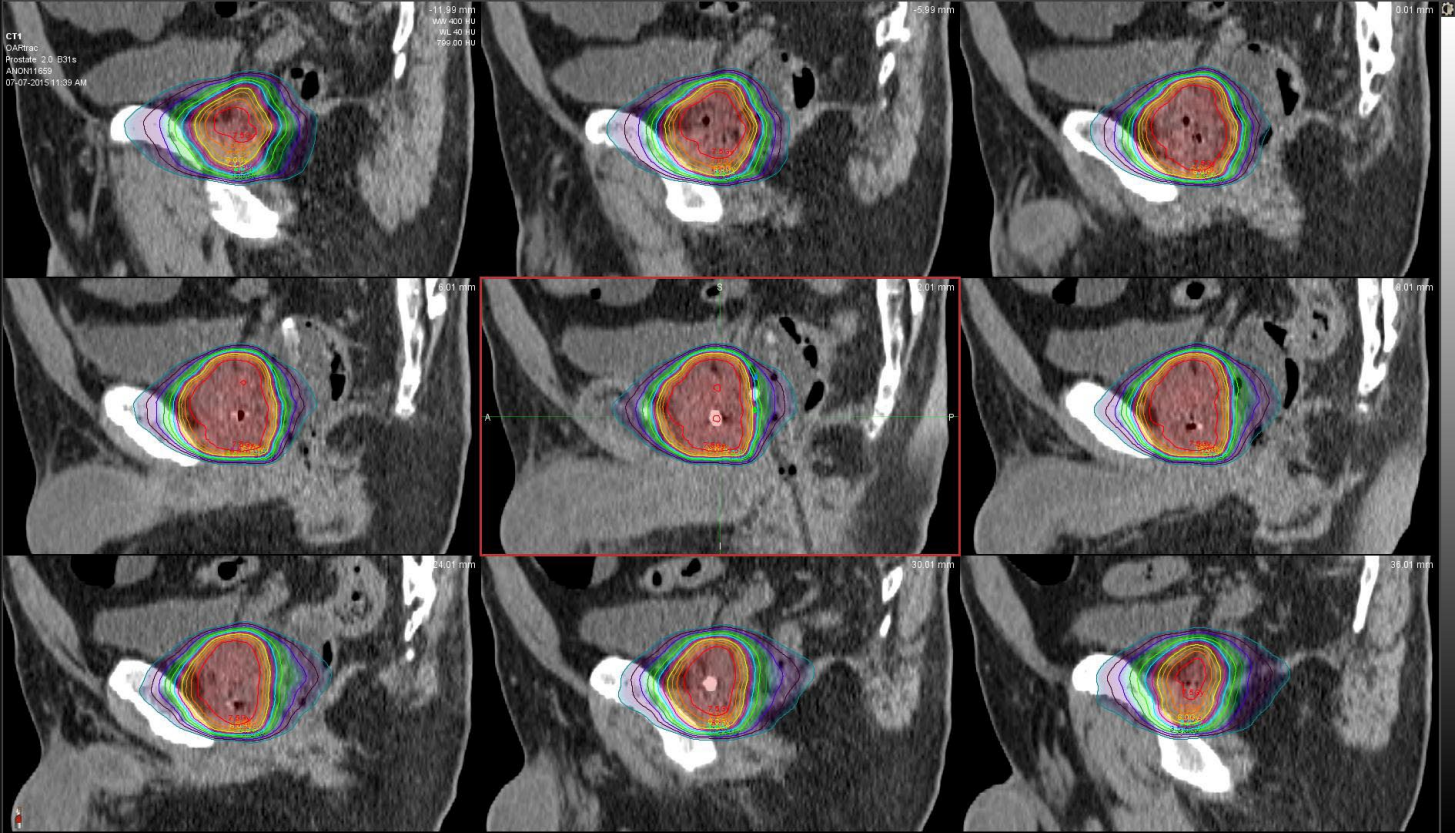
Sagittal view of the prostate with isodose lines for one fraction of the SBRT treatment. Estimated positions of the proximal and distal detectors can be seen in the central image. Each isodose line represents a change of 50 cGy. Slices moving patient left to patient right from top left to bottom right.

The measured doses were compared to the expected doses from the treatment planning software and the fraction‐specific doses from the MIM software. The expected doses were found in two ways: first, expected doses were found in the treatment planning system using only the treatment planning CT and, second, expected doses were found in MIM using the CBCT of each individual fraction. If daily CBCT images were not available, expected doses would only be available from the treatment planning system using the treatment planning CT. The results are summarized in Tables 1 and 2. While the average measured dose at the proximal detector was 431.9 cGy as compared to 458.0 cGy calculated dose (about 6% below predicted value) and the average measured dose at the distal detector was 512.9 cGy as compared to the calculated dose of 456.7 cGy (12.3% higher dose value than expected value), the overall average of measured dose difference was 6% of predicted dose. Thus it correlates well with the average detected doses in the solid‐water phantom. The measured daily doses showed a wide range of agreement with the expected doses, and the main reason was believed to be positioning uncertainty. Other causes for discrepancy certainly could affect the readings as well. While the positional change in the endorectal balloon was the most likely cause, patient positioning and alterations in the delivered dose due to inhomogeneous tissue density within the adjacent region of the detector such as bowel gas might also have contributed. Additionally the very steep drop‐off of dose with SBRT compared with standard radiotherapy accentuates the difference. A HexaPOD couch (Elekta, Stockholm, Sweden) was used for fiducial alignment to correct for tilt of the pelvis to help minimize uncertainty and to align the prostate to the planning position prior to the start of treatment. A total of three CBCT scans were taken with one prior to the treatment, one between the two treatment arcs, and one after the treatment to account for positional uncertainty during treatment as best as able. For SBRT treatments, the dose gradient is high at the periphery of the target volume and a difference of a few millimeters can result in large changes in dose (see Figs. 2 and 3). Using the MIM software, it was possible to find the distance to agreement (DTA), which was the shortest distance from the estimated location of the PSD to the location that had the exact same calculated dose as the dose measured by the PSD. Results are shown in Table 3.

**Table 1:** Measured and expected doses for the proximal detector. All doses are in cGy

	*Fraction 1*	*Fraction 2*	*Fraction 3*	*Fraction 4*	*Fraction 5*	*Total*
Measured Dose	417.11	603.90	425.91	291.71	420.66	2159.29
Pinnacle Dose	458	458	458	458	458	2290
% Difference	−8.93%	+31.86%	−7.01%	−36.31%	−8.15%	−5.71%
MIM Dose	531	399	497	395	474	2296
% Difference	−21.45%	+51.35%	−14.30%	−26.15%	−11.25%	−5.95%

**Table 2:** Measured and expected doses for the distal detector. All doses are in cGy

	*Fraction 1*	*Fraction 2*	*Fraction 3*	*Fraction 4*	*Fraction 5*	*Total*
Measured Dose	433.25	323.17	593.22	692.72	521.98	2564.34
Pinnacle Dose	456.7	456.7	456.7	456.7	456.7	2283.5
% Difference	−5.13%	−29.24%	+29.89%	+51.68%	+14.29%	+12.30%
MIM Dose	429	407	435	549	457	2277
% Difference	+0.99%	−20.60%	+36.37%	+26.18%	+14.22%	+12.62%

**Table 3:** Distance to agreement for the two PSD detectors for each treatment fraction

	*Fraction 1*	*Fraction 2*	*Fraction 3*	*Fraction 4*	*Fraction 5*
DTA – Proximal Detector (mm)	4.5	5.0	2.5	3.5	2.0
DTA – Distal Detector (mm)	0.6	9.0	4.5	4.0	2.5

## IV. DISCUSSION

Positional uncertainty errors may be variable even beyond the difference between the CBCT and planning CT due to possible intrafraction prostate motion occurring between setup and completion of the dose delivery for the SBRT. While the use of the endorectal balloon component of this device greatly limits prostate motion, patient immobilization is unable to completely eliminate patient motion due to skeletal muscle fatigue or discomfort from setup immobilization and the table surface causing the patient to make voluntary adjustments in their positioning, or due to gas or bladder or bowel motion. Due to the high‐dose gradients associated with SBRT treatments, positional uncertainties of a few millimeters can lead to large differences between measured and expected doses. Because the DTA points are usually within a few millimeters, this is the likely source of the differences seen for this patient. Better localization of the PSDs in CT scans, for example using a modified sensor with more radiopaque markers (the current sensor currently contains only one between the PSDs), could improve the accuracy in identifying the PSDs in the CT scans, and ultimately could lead to improvement in the *in vivo* dosimetry measurements.

## V. CONCLUSIONS

While this manuscript is exploratory in reporting the first ever use of this novel device in a patient for treatment delivery using SBRT to treat prostate cancer, the clinical implications are very pertinent to improving patient care. By providing an *in vivo* reading of actual delivered daily dose, it may help to reduce treatment errors in daily setup or initial dose calculations. Patient safety and treatment efficacy are improved through the use of the technology, especially in the setting of hypofractionated treatments, where a daily error can result in a larger deviation in total delivered dose. We are now utilizing this technology in a novel partial prostate SBRT protocol and should anticipate the ability to provide further updates in patient reported outcomes. This is the first reported case using both a hydrogel and the *in vivo* dosimetry system with PSDs and ERB to maximally reduce dose to the rectal wall and minimize prostate motion during SBRT to reduce late rectal toxicity. This will now be further clinically evaluated on an IRB‐approved prospective study in 12 patients using OARtrac system without a hydrogel device. The goal of this study will be to treat a limited volume of the prostate gland as defined through a combination of both anatomic and functional MRI sequencing and correlated with tracked histopathological evaluation in and around the index lesion to define a planning target volume for a 3‐fraction regimen of SBRT of 9.75 Gy per fraction to a total dose of 29.25 Gy. This trial will be using a quality of life endpoint to evaluate treatment tolerance and side effects in addition to biochemical response with PSA and serial MRI imaging. The use of OARtrac system in this study was initiated as a result of a recommendation by the protocol review and monitoring committee to track daily delivered dose to assure patient safety and will be correlated to patient reported toxicities. The results from this trial will be the subject of a future manuscript.

## ACKNOWLEDGMENTS

Dr. Justin Cantley and Dr. Rodney Ellis each reports that he has a consulting agreement with the company RadiaDyne to provide clinical advice as needed. Dr. Ellis also reports that he was paid a small fee (<$2000) to provide a dinner talk for RadiaDyne at ASTRO 2015. In addition, this technology will be used in protocol for partial prostate SBRT treatments of which Dr. Ellis is the co‐inventor. The device and equipment were provided by RadiaDyne for the use of this case study and the partial prostate SBRT treatments, and the publication fee was also paid by RadiaDyne.

## COPYRIGHT

This work is licensed under a Creative Commons Attribution 3.0 Unported License.
